# Induction of Apoptosis by Fucoidan in Human Leukemia U937 Cells through Activation of p38 MAPK and Modulation of Bcl-2 Family

**DOI:** 10.3390/md11072347

**Published:** 2013-07-04

**Authors:** Hyun Soo Park, Hye Jin Hwang, Gi-Young Kim, Hee-Jae Cha, Wun-Jae Kim, Nam Deuk Kim, Young Hyun Yoo, Yung Hyun Choi

**Affiliations:** 1 Department of Pharmacy, Pusan National University, Busan 609-735, Korea; E-Mails: phsvic@naver.com (H.S.P.); nadkim@pusan.ac.kr (N.D.K.); 2 Department of Food and Nutrition, Dongeui University, Busan 614-714, Korea; E-Mail: hhj2001@deu.ac.kr; 3 Anti-Aging Research Center & Blue-Bio Industry Regional Innovation Center, Dongeui University, Busan 614-714, Korea; 4 Department of Marine Life Sciences, Jeju National University, Jeju 690-756, Korea; E-Mail: immunkim@cheju.ac.kr; 5 Departments of Parasitology and Genetics, Kosin University College of Medicine, Busan 602-702, Korea; E-Mail: hcha@kosin.ac.kr; 6 Department of Urology, Chungbuk National University College of Medicine, Cheongju 361-763, Korea; E-Mail: wjkim@chungbuk.ac.kr; 7 Department of Anatomy and Cell Biology, College of Medicine, Dong-A University, Busan 602-714, Korea; 8 Department of Biochemistry, Dongeui University College of Oriental Medicine, Busan 614-052, Korea

**Keywords:** fucoidan, leukemic cells, apoptosis, p38 MAPK, Bcl-2

## Abstract

The present study investigated possible mechanisms on the apoptosis induction of human leukemic cells by fucoidan, a sulfated polysaccharide found in marine algae. Fucoidan treatment of cells resulted in inhibition of growth and induction of apoptosis, as measured by 3-(4,5-dimetylthiazol-2-yl)-2,5-diphenyl-tetrazolium (MTT) assay, fluorescence microscopy, DNA fragmentation, and flow cytometry analysis. The increase in apoptosis was associated with the proteolytic activation of caspases, Bid cleavage, insertion of pro-apoptotic Bax into the mitochondria, release of cytochrome *c* from mitochondria to cytosol, and loss of mitochondria membrane potential (MMP) in U937 cells. However, apoptosis induced by fucoidan was attenuated by caspase inhibitors, indicating that fucoidan-induced apoptosis was dependent on the activation of caspases. Furthermore, fucoidan treatment effectively activated the p38 mitogen-activated protein kinase (MAPK) and p38 MAPK inhibitor, SB203580, and significantly reduced fucoidan-induced apoptosis through inhibition of Bax translocation and caspases activation, suggesting that the activation of p38 MAPK may play a key role in fucoidan-induced apoptosis. In addition, the authors found fucoidan-induced significantly attenuated in Bcl-2 overexpressing U937 cells, and pretreatment with fucoidan and HA 14-1, a small-molecule Bcl-2 inhibitor, markedly increased fucoidan-mediated apoptosis in Bcl-2 overexpressing U937 cells. Our findings imply that we may attribute some of the biological functions of p38 MAPK and Bcl-2 to their ability to inhibit fucoidan-induced apoptosis.

## 1. Introduction

Apoptosis, a programmed cell death, plays a fundamental role in the normal development and differentiation of multicellular organisms. Apoptosis also occurs as a reaction to protect cells damaged by diseases or noxious agents. The two types of apoptosis include an extrinsic pathway that involves transmembrane death receptor-mediated interactions and an intrinsic pathway that involves mitochondria-mediated stimuli [1]. Interaction between ligands and death receptors initiates the extrinsic pathway at the plasma membrane and subsequently the activation of caspase-8. Caspase-8, an initiator caspase, can directly activate downstream effector caspases, including caspase-3 [2,3]. In some cells, caspase-8 also mediates the intrinsic pathway via cleavage of the pro-apoptotic Bid, a BH3-only protein [4,5]. A broad range of physical and chemical stimuli cause mitochondrial dysfunction, which triggers the intrinsic pathway [6,7]. Mitochondrial dysfunction induces activation of caspase-9 and subsequently activates effector caspases, such as caspase-3. Following activation of caspase-3, cleavage of several specific substrates occurs, including poly(ADP-ribose) polymerase (PARP), eventually leading to apoptosis [8]. Because many chemopreventive and chemotherapeutic agents can cause cell death via induction of apoptosis, induction of apoptotic cell death represents an important mechanism in the anti-cancer properties of many drugs. 

Mitogen-activated protein kinases (MAPKs), members of the serine/threonine kinase family, including c-Jun NH_2_-terminal kinase (JNK), extracellular signal-regulated kinase (ERK), and p38 MAPK are activated in response to various stimuli and participate in a variety of signaling pathways that regulate diverse cellular processes including cell growth, differentiation, and stress responses. Activation of MAPKs closely relates to apoptosis induced by stress stimuli [9,10,11]. Among them, the p38 MAPK pathway becomes activated in a wide variety of cancers and results in enhanced resistance to apoptosis through multiple mechanisms [12,13]. Thus, inhibition of p38 MAPK can decrease cell survival and enhance the effects of chemotherapeutic drugs in many types of cancer cells.

Fucoidan, a sulfated polysaccharide found in brown algae, such as *Fucus vesiculosus* and *Cladosiphon okamuranus*, contains considerable amounts of l-fucose and sulfate esters [14,15], and possesses a variety of biological activities including anti-viral, anti-microbial, and anti-inflammatory effects [16,17,18]. This marine natural product (in a pure, semi-pure or extract form) is available as a dietary supplement and is consumed for health benefits in many countries. Previous reports also indicated that fucoidan has exhibited anti-cancer properties by inducing cell cycle arrest and apoptosis in several types of human cancer cells* in vitro* [19,20,21,22,23,24]. However, researchers have yet to completely understand cellular and molecular mechanisms underlying the compound. Thus, the present study investigated the mechanisms of fucoidan-induced apoptosis in human leukemic cells. Our results demonstrated that crude fucoidan, isolated from *Fucus vesiculosus*, triggers apoptosis of U937 cells through activation of the intrinsic caspase pathway along with the death receptor-mediated extrinsic pathway, accompanied by activation of p38 MAPK.

## 2. Results and Discussion

### 2.1. Fucoidan Inhibits Cell Growth and Induces Apoptosis in Leukemic Cells

**Figure 1:**
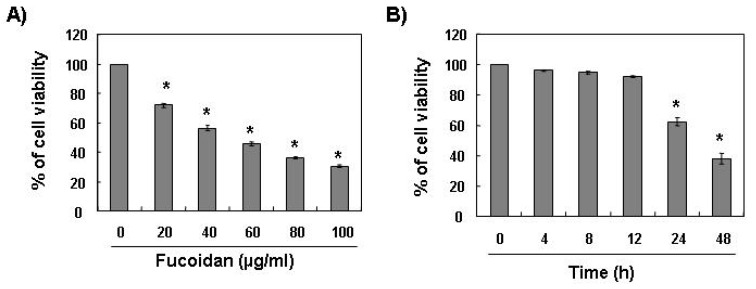
Effects of fucoidan on cell viability in U937 cells. U937 cells were plated at a concentration of 2.5 × 10^5^ cells in 6-well plates. Following 24 h of stabilization, cells were treated with the indicated concentrations of fucoidan for 48 h (**A**) or 80 μg/mL of fucoidan for the indicated times (**B**). The cell viability was measured by the metabolic-dye-based MTT assay. Results are expressed as percentage of the vehicle treated control ± standard deviation (SD) of three separate experiments. The significance was determined by Student’s *t*-test (*p < 0.05* vs.* untreated control).

To investigate the effect of fucoidan on cell growth of leukemic cells, U937 cells were exposed to various concentrations of fucoidan for 48 h or 80 μg/mL of fucoidan for the various times points, and cell viability was then measured by the MTT assay. As shown in Figure 1, treatment with fucoidan decreased the viability of U937 cells in a concentration- and time-dependent manner. 

**Figure 2:**
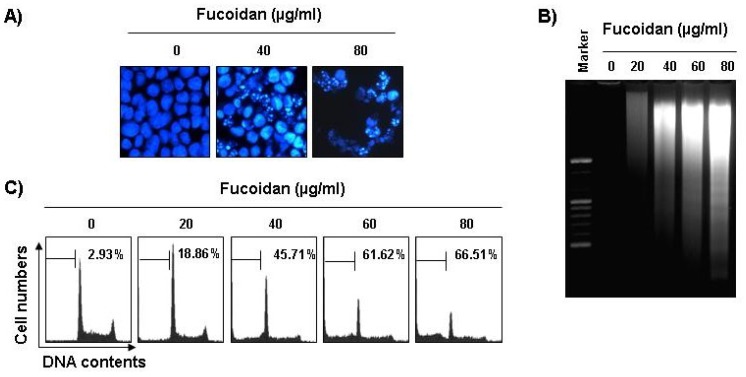
Induction of apoptosis by fucoidan treatment in U937 cells. (**A**) Following 24 h of stabilization, cells were incubated with various concentrations of fucoidan for 48 h. The cells were fixed and stained with DAPI solution. The stained nuclei were then observed under a fluorescent microscope (×400); (**B**) For the analysis of DNA fragmentation, genomic DNA from cells was extracted, separated by 2.0% agarose gel electrophoresis, and visualized under UV light after staining with EtBr. Marker indicates a size marker of the DNA ladder; (**C**) To quantify the degree of apoptosis induced by fucoidan, cells were evaluated by flow cytometry for sub-G1 DNA content (hypodiploid DNA), which represents the cells undergoing apoptotic DNA degradation. Data are the mean ± SD of two different experiments.

The next experiments were performed to determine if this inhibitory effect of fucoidan on cell viability resulted from apoptotic cell death. To examine apoptosis morphologically, the nuclei of untreated and fucoidan-treated cells were stained with 4,6-diamidino-2-phenyllindile (DAPI) solution and then observed. The control cells displayed intact nuclear structure while cells treated with fucoidan had apoptotic morphological characteristics, such as chromatin condensation and nuclear fragmentation in U937 cells (Figure 2A). In addition, nucleosomal DNA ladder formation by agarose gel electrophoresis was observed in U937 cells treated with over 40 μg/mL of fucoidan for 48 h (Figure 2B). We further quantified the degree of apoptotic dead cells by cell cycle analysis. As indicated in Figure 2C, fucoidan treatment resulted in a significantly increased accumulation of U937 cells at the apoptotic sub-G1 phase and that this response occurred in a concentration-dependent manner. 

**Figure 3:**
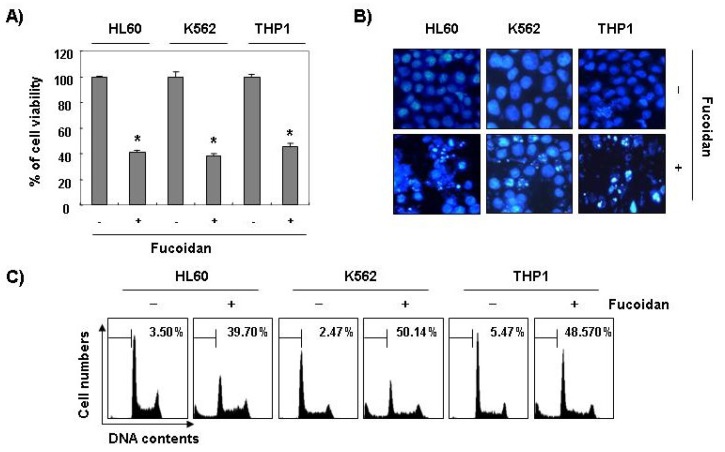
Inhibition of cell viability and induction of apoptosis by fucoidan in other leukemic cells. Three leukemic cell lines (HL60, K562, and THP1) were treated with 80 μg/mL fucoidan for 48 h. (**A**) The cell viability was measured by the metabolic-dye-based MTT assay. Each point represents the mean ± SD of three independent experiments. The significance was determined by Student’s *t*-test (*p < 0.05* vs.* untreated control); (**B**) The cells were stained with DAPI solution and stained nuclei were then observed under a fluorescent microscope (×400); (**C**) The percentage of cells with hypodiploid DNA (sub-G1 phase) were measure by flow cytometry. Each point represents the mean of two independent experiments.

Furthermore, fucoidan significantly inhibited cell viability and induced apoptosis in other leukemic cell lines, such as HL60, K562, and THP1 (Figure 3). These results demonstrated an association between the growth inhibition observed in response to fucoidan and the induction of apoptosis in leukemic cells.

### 2.2. Fucoidan Induces Activation of Caspases and Inhibits the Levels of IAP Family Proteins in U937 Cells

**Figure 4:**
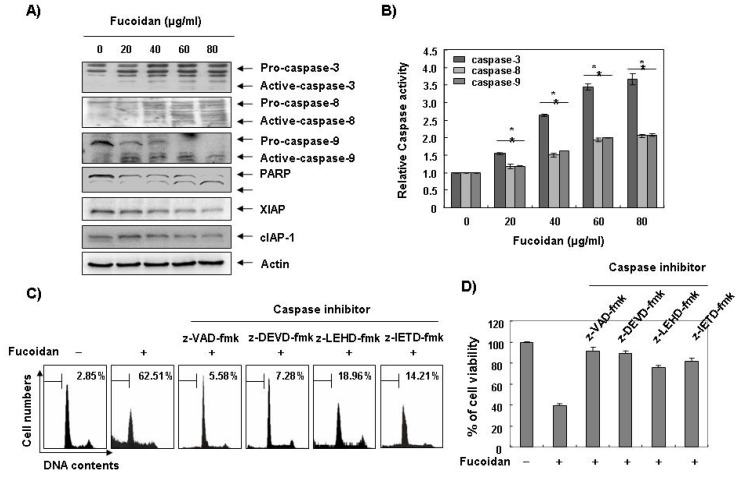
Activation of caspases, degradation of PARP, and inhibition of IAP family proteins by fucoidan in U937 cells. (**A**) U937 cells were treated with the indicated concentrations of fucoidan for 48 h. The cells were lysed and then cellular proteins were separated by sodium dodecyl sulfate (SDS)-polyacrylamide gels and transferred onto nitrocellulose membranes. The membranes were probed with the indicated antibodies. Proteins were visualized using an enhanced chemiluminescence (ECL) detection system. Actin was used as an internal control; (**B**) After 48 h incubation with the indicated concentrations of fucoidan, the cells were lysed and aliquots (50 μg protein) were assayed for* in vitro* caspase-3, -8, and -9 activity using DEVD-pNA, IETD-pNA, and LEHD-pNA as substrates, respectively, at 37 °C for one hour. The released fluorescent products were measured. Data are expressed as mean ± SD of three independent experiments. The significance was determined by Student’s *t*-test (*p < 0.05* vs.* untreated control); (**C** and **D**) The cells were incubated with or without 80 μg/mL fucoidan for 48 h after one hour pretreatment with or without the indicated caspase specific inhibitors (50 μM; z-VAD-fmk, pan-caspase inhibitor; z-DEVD-fmk, caspase-3 inhibitor; z-LEHD-fmk, caspase-9 inhibitor and z-IETD-fmk, caspase-8 inhibitor) and then DNA contents were analyzed by a flow cytometer (**C**). Each point represents the average of two independent experiments. The degree of growth inhibition was determined by MTT assay (**D**).

Caspases, known to serve as important mediators of apoptosis in both intrinsic and extrinsic pathway, also contribute to general apoptotic morphology through the cleavage of various cellular substrates, including PARP. Therefore, to gain further insight into the mechanism by which fucoidan induces apoptosis we examined the effects of fucoidan on caspase protein levels and their activities as well as their inhibitor proteins, inhibitor of apoptosis proteins (IAP) family proteins. As Figure 4A,B reveals, Western blot analyses showed that fucoidan treatment induced an increase in the levels of active-caspase-3, -8, and -9 proteins, and their activities in a concentration-dependent manner. Subsequent Western blot analysis revealed that progressive proteolytic cleavage products of PARP protein and accumulation of the 85 kDa, a downstream target of the activated caspase-3 [8], occurred in U937 cells treated with fucoidan. In order to demonstrate that the activation of caspases is a key step in the apoptotic pathway induced by fucoidan, U937 cells were pretreated with a pan-caspase inhibitor (z-VAD-fmk) and potential caspase-specific inhibitors (z-DEVD-fmk, z-IETD-fmk, and z-LEHD-fmk for the inactivation of caspase-3, -8, and -9, respectively) for one hour, followed by treatment with fucoidan for 48 h. As shown in Figure 4C, pretreatment with caspase inhibitors significantly blocked the increase in the sub-G1 population and restored the decreased viability induced by fucoidan (Figure 4C,D). These results indicate that fucoidan treatment induces apoptosis in U937 cells through a caspase-dependent pathway. Furthermore, fucoidan treatment down-regulated the levels of IAP family proteins, such as XIAP and cIAP-1 (Figure 4A), which bind to caspases and lead to their inactivation [25]. These results indicate that fucoidan treatment induces apoptosis through activation of caspases and reduction of IAP family proteins in U937 cells. 

### 2.3. Fucoidan Induces Loss of Mitochondrial Membrane Potential (MMP) and Modulation of Bcl-2 Family Members in U937 Cells

**Figure 5:**
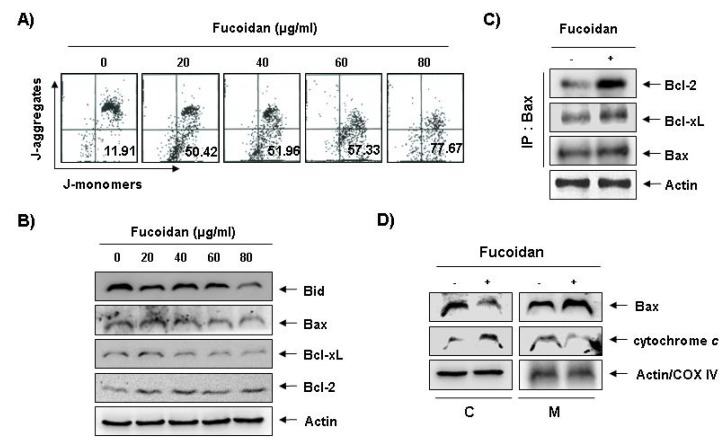
Effects of fucoidan on levels of mitochondria membrane potential (MMP) values and Bcl-2 family proteins, and Bax translocation to mitochondria in U937 cells. (**A**) Ollowing 24 h of stabilization, U937 cells were treated with the indicated concentrations of fucoidan for 48 h. Cells were collected and incubated with JC-1 (10 μM) for 20 min at 37 °C in the dark. The cells were the washed once with phosphate buffered saline (PBS) and analyzed by a DNA flow ctometer. The results are expressed as the mean of two independent experiments; (**B**) The cell lysates obtained from cells grown under the same conditions as (**A**) were separated by SDS-polyacrylamide gels and transferred onto nitrocellulose membranes. The membranes were probed with the indicated antibodies. The proteins were visualized using an enhanced chemiluminescence (ECL) detection system; (**C**) After treatment with or without 80 μg/mL fucoidan for 48 h, total cell lysates were immunoprecipitated with anti-Bax antibody, separated on 10% SDS-polyacrylamide gels, and transferred to nitrocellulose. The levels of Bcl-2 and Bcl-xL proteins were detected with anti-Bcl-2 and anti-Bcl-xL antibodies, respectively, and ECL detection. Immunoprecipitation (IP) actin was used as an internal control; (**D**) The mitochondrial (M) and cytosolic (C) proteins were extracted and analyzed by Western blotting using the indicated antibodies. Actin and cytochrome oxidase 4 (COX4) were used as internal controls for the cytosolic and mitochondrial fractions, respectively.

To investigate whether fucoidan-induced apoptosis in U937 cells involves mitochondrial pathway, we next examined the levels of MMP values and Bcl-2 family proteins by a flow cytometer after staining with 5,5′,6,6′-tetrachloro-1,1′,3,3′-tetraethyl-imidacarbocyanine iodide (JC-1), a fluorescent cationic dye, and Western blot analysis, respectively. As Figure 5A shows, fucoidan treatment caused a concentration-dependent loss of MMP compared with untreated control. In addition, although we did not detect the truncated form of pro-apoptotic protein Bid, fucoidan decreased the whole form of Bid proteins (Figure 5B). In addition, while the level of anti-apoptotic Bcl-xL protein decreased concentration-dependency in fucoidan-treated U937 cells, anti-apoptotic Bcl-2 and pro-apoptotic Bax proteins, which function as critical proteins to maintain the stabilization of mitochondria, slightly increased and decreased in response to fucoidan treatment, respectively (Figure 5B). This result signified an opposite outcome to that found in other studies, because researchers generally believe that Bax increases during the apoptotic process. Further, by and large, we have known Bcl-2 protein to inhibit apoptosis by binding with pro-apoptotic Bax in mitochondria. Therefore, the authors additionally investigated whether or not Bax translocated from cytosol to mitochondria after fucoidan treatment by Co-IP assay and mitochondria fraction. As indicated in Figure 5C, fucoidan treatment significantly increased the interactions between Bax and Bcl-2, as well as Bax and Bcl-xL. Significantly, we found the binding between Bax and Bcl-2 stronger than that of Bax and Bcl-xL. Furthermore, fucoidan treatment also decreased cytosolic levels of Bax and increased cytosolic levels of cytochrome *c*, while mitochondrial levels of Bax significantly increased, and mitochondrial levels of cytochrome *c* significantly decreased, respectively (Figure 5 D). These results suggest that fucoidan inserts Bax from cytosol into mitochondria inducing increased binding between Bax and Bcl-2, and loss of MMP resulting in mitochondrial dysfunction, release of cytochrome *c* to cytosol and apoptosis induction.

### 2.4. Activation of p38 Mitogen-Activated Protein Kinase (MAPK) is Involved in Fucoidan-Induced Apoptosis in U937 Cells

**Figure 6:**
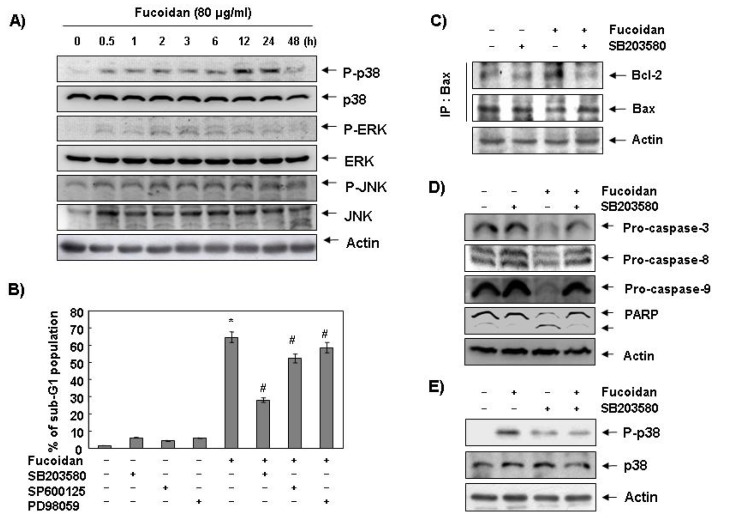
Effects of p38 MAPK activation on fucoidan-induced apoptosis in U937 cells. (**A**) Cells were treated with fucoidan (80 μg/mL) for the indicated times. Cells were then lysed, and equal amounts of cell lysates were resolved by SDS-polyacrylamide gels, transferred to nitrocellulose, and probed with the indicated antibodies; (**B**) Cells were pretreated with the indicated MAPK inhibitors (SB203580 (10 μM), SP600125 (40 μM) and PD98059 (100 μM)) for one hour and then treated with fucoidan (80 μg/mL) for 48 h. The percentage of sub-G1 population was evaluated by a flow cytometer. Each point represents the mean ± SD of three independent experiments. The significance was determined by Student’s *t*-test (*, *p* < 0.05* vs.* untreated control; ^#^, *p* < 0.05 present* vs.* absent MAPK inhibitors); (**C**) Cells were pretreated with SB203580 (10 μM) for one hour before treatment with 80 μg/mL of fucoidan for 48 h. Bax proteins were immunoprecipitated using ant-Bax antibody, and the immunoprecipitated proteins were separated on SDS-polyacrylamide gels and transferred to nitrocellulose membranes for Western blot analysis using anti-Bcl-2 and Bax antibodies; (**D**) Equal amounts of cell lysates extracted from cells were resolved by SDS-polyacrylamide gels and transferred to nitrocellulose membranes. The membranes were probed with the indicated antibodies and the proteins were visualized using an ECL detection system; (**E**) Cells were pretreated with 10 μM SB203580 for one hour and then treated with 80 μg/mL fucoidan for 24 h. They were then lysed, and equal amounts of cell lysates were resolved by SDS-polyacrylamide gels, transferred to nitrocellulose, and probed with the indicated antibodies. Actin was used as an internal control.

Next, we investigated the effect of fucoidan treatment on the expression and activities of MAPKs to determine if these signaling pathways play a role in mediating the observed apoptotic response. As Figure 6A demonstrates, the phosphorylated levels of p38 MAPK proteins significantly increased after 12 h and 24 h treatment of fucoidan, compared with ERK and JNK. To confirm an association between the activation of p38 MAPK and the apoptosis induction by fucoidan, we pretreated the cells with MAPK inhibitors and analyzed the sub-G1 DNA content by a flow cytometer. As shown in Figure 6B, pretreatment with SB203589 (a specific inhibitor of p38 MAPK) significantly reduced the increased number of cells with the sub-G1 DNA content by fucoidan. However, pretreatment with PD98059 (a potent inhibitor of ERK) or SP600125 (a potent inhibitor of JNK) did not have a significant effect on the fucoidan treatment indicating a close involvement of the activation of p38 MAPK with fucoidan-induced apoptosis in U937 cells.

### 2.5. Fucoidan-Induced Activation of p38 MAPK Causes Bax Translocation to Mitochondria in U937 Cells

To further determine the mechanism of p38 MAPK activation by fucoidan in U937 cells, we investigated the binding between Bcl-2 and Bax and expression levels of caspases. As Figure 6C unveils, pretreatment with p38 MAPK-specific inhibitor significantly decreased fucoidan-increased binding between Bcl-2 and Bax in U937 cells. Inhibition of p38 MAPK also recovered fucoidan-induced reduction of pro-caspase-3, -8, and -9, cleavage of PARP and phosphorylation of p38 MAPK (Figure 6D,E). These results suggest that fucoidan-induced activation of p38 MAPK leads to apoptosis by activation of caspases via Bax translocation from cytosol to mitochondria.

### 2.6. Fucoidan-Induced Apoptosis is Suppressed in Bcl-2 Overexpressing U937 Cells and by HA14-1, Bcl-2 Inhibitor

**Figure 7:**
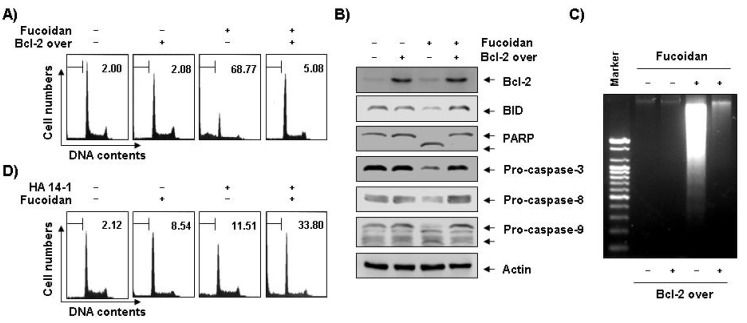
Effects of Bcl-2 overexpression and Bcl-2 inhibitor on fucoidan-induced apoptosis in U937 cells. (**A**–**C**) Control (−, U937/vector) or Bcl-2 transfected (+, U937/Bcl-2) cells were incubated with 80 μg/mL of fucoidan for 48 h. The cells were collected, DNA contents were analyzed by a flow cytometer (**A**), and the fragmented DNA was extracted and analyzed on a 2.0% agarose gel containing EtBr (**C**). (**B**) Equal amounts of cell lysate were extracted, resolved on SDS-polyacrylamide gels, transferred to nitrocellulose membranes, and probed with the indicated antibodies. The proteins were visualized using an ECL detection system. Actin was used as an internal loading control. (**D**) Bcl-2 overexpressing U937 cells were treated with 80 μg/mL of fucoidan in the presence and absence of 15 μM of HA14-1 for 48 h. The percentage of cells with hypodiploid DNA (sub-G1 phase) content represent the fractions undergoing apoptotic DNA degradation. Each point represents the mean of two independent experiments.

To determine the role that Bcl-2 plays in fucoidan-induced apoptosis, U937 cells were stably transfected with either human Bcl-2 cDNA (U937/Bcl-2) or vector alone (U937/vector). G418-resistant clones found to overexpress Bcl-2 proteins were then selected and used for subsequent experiments. Our results indicate that Bcl-2 overexpression significantly protects cells from fucoidan-induced formation of cells in the sub-G1 population and DNA fragmentation (Figure 7A,B) when compared to U937/vector cells. Additionally, fucoidan treatment in Bcl-2 overexpressing U937 cells fully recovered the fucoidan-induced reduction of caspases (-3, -8, and -9) and Bid, and cleavage of PARP (Figure 7C). Although the total levels of Bcl-2 proteins slightly increased due to fucoidan treatment, these results suggest that the overexpression of anti-apoptotic Bcl-2 protein attenuates fucoidan-induced apoptosis. To find other evidence of the importance of Bcl-2 in fucoidan-induced apoptosis in U937 cells, we next determined the ability of a small molecule antagonist for Bcl-2, HA14-1, to reverse the effects of Bcl-2 on fucoidan-mediated apoptosis. As shown in Figure 7D, co-administration of fucoidan and HA14-1 synergistically increased sub-G1 population from 8.5% to 33.8% in fucoidan-treated Bcl-2 overexpressing U937 cells, meaning that Bcl-2 plays critical roles in fucoidan-induced apoptosis in U937 cells.

### 2.7. Global Discussion

Although findings from recent studies have demonstrated that fucoidan, a sulfated polysaccharide found in brown algae, can suppress the growthof various cultured human cancer cell lines* in vitro* [19,20,21,22,23,24], the signaling pathway by which this occurs remains unclear. The present study aimed to determine the capacity of fucoidan to induce apoptosis and to identify the related biochemical mechanisms in human leukemic cells. The present results clearly demonstrate that fucoidan inhibits leukemic cell growth by induction of apoptotic cell death, which appears to account for its anti-proliferating activity. Measurement of chromatin condensation of the nuclei, DNA fragmentation by agarose gel electrophoresis, and induction of sub-G1 phase by flow cytometry analysis confirmed induction of apoptosis by leukemic cells (Figure 1, Figure 2, Figure 3). 

Apoptosis plays an important role in the normal development and differentiation of multicellular organisms, characterized by morphological and biological changes such as cytoplasmic shrinkage, chromatin condensation, and DNA degradation [1]. Apoptosis also serves as a critical protective mechanism against carcinogenesis caused by mutations of genetic materials of normal cells or various carcinogens [26]. A variety of stimuli can trigger it, including death receptor-mediated signaling (extrinsic pathway) or intracellular stresses (intrinsic pathway) [1,27]. Because alterations in mitochondria structure and function, as well as activation of caspase family that has critical roles in regulating apoptosis, initiate apoptosis, we first investigated the catalytic activity of caspases and mitochondrial dysfunction to gain insight into the process of fucoidan-induced apoptosis. The present data revealed that fucoidan reduced the levels of pro-caspase-8 and -9, and increased their catalytic activity, which involves initiator caspases of extrinsic and intrinsic pathways, respectively, in U937 cells (Figure 4). Fucoidan also down-regulated IAP family proteins, such as XIAP and cIAP-1 (Figure 4), which reportedly block apoptosis due to their function as direct inhibitors by binding to and inhibiting several caspases. Researchers have considered them to cause resistance to apoptosis in cancer [25,28]. Furthermore, fucoidan markedly induced the loss of MMP, an important parameter of mitochondrial function used as an indicator of cell condition, and activated the key executioner, caspase-3, and concomitant degradation of PARP (Figure 4, Figure 5). However, blocking caspase activity by pretreating the cells with a pan-caspase inhibitor and caspase specific inhibitors, significantly prevented fucoidan-induced apoptosis and growth inhibition (Figure 4). Therefore, the data suggest that fucoidan-induced apoptosis in both the U937 cells is caspase-dependent, and the apoptotic effects of fucoidan appear to involve activation of both the intrinsic and extrinsic pathways.

In the mitochondrial pathway, the ratio of expression of the pro-apoptotic proteins such as Bax and the anti-apoptotic proteins such as Bcl-2 and Bcl-xL ultimately determines cell death or survival through regulation of mitochondrial permeability transition. In addition, caspase-8 mediates the intrinsic pathway via cleavage of the pro-apoptotic Bid protein, a BH3-only protein, to a truncated Bid (tBid) through translocation from the cytosol to the mitochondria, triggering mitochondrial dysfunction, followed by activation of caspase-9. This leads to activation of caspase-3 for induction of apoptosis via a release of cytochrome *c* to cytosol after translocation of tBid to the mitochondria [4,29,30]. Translocation of pro-apoptotic Bax proteins from cytosol to mitochondria also represents a key event for the activation of apoptosis. The inserted Bax in mitochondria exists as dimers or oligomers, whereas in the cytosol Bax exists as monomers [31], and the release of cytochrome *c* requires mitochondrial membrane insertion and oligomerization of Bax [32]. Our results indicated that fucoidan treatment induced reduction of whole Bid proteins, which may relate to the activation of tBid (Figure 5B). Results from Co-IP and mitochondria fraction assays demonstrated that the binding activity between Bcl-2 and Bax proteins, and the levels of Bax proteins in mitochondria and the release of cytochrome *c* from mitochondria to cytosol increased (Figure 5C,D). The data clearly indicate that fucoidan induced Bax translocation to mitochondria from cytosol, leading to the release of cytochrome *c*, apoptosome formation, and finally induction of apoptosis in U937 cells. 

On the other hand, the MAPKs including, for example, ERK, JNK, and p38 MAPK play critical roles in cell survival and apoptosis in various cancer cells. We know that the activation of the p38 MAPK and JNK pathways leads to induction of apoptosis, whereas we more often associate the ERK with cell survival [33,34]. Notably, researchers have reported that p38 MAPK regulates the translocation of Bax from cytosol to the mitochondria in response to a variety of stimuli leading to an apoptotic cell death [35]. The data mean that activation of p38 MAPK can induce mitochondrial dysfunction and subsequently release apoptogenic proteins such as cytochrome *c* from the mitochondria to cytosol, and finally caspase-9 and -3 become activated. In this report, treatment with fucoidan resulted in up-regulation of the p38 MAPK phosphorylation, as opposed to ERK and JNK signaling pathways. Therefore, we investigated the involvement of activation of p38 MAPK signaling pathway in fucoidan-induced apoptosis in U937 cells. As the results indicate, SB203580, specific inhibitor of p38 MAPK, markedly inhibited fucoidan-induced apoptosis of U937 cells by inhibiting the interaction between Bcl-2 and Bax and caspases activation (Figure 6) suggesting an association of fucoidan-induced apoptosis with Bax translocation to mitochondria via activation of p38 MAPK.

Interestingly, our results indicate pro-apoptotic Bax and anti-apoptotic Bcl-2 levels slightly decreased and increased in response to fucoidan treatment, respectively (Figure 5B). However, through mitochondrial fractionation assay, we observed that the levels of Bax proteins increased in the mitochondria and decreased in the cytosol, while cytochrome *c* represented the reverse tendency (Figure 5D), which correlated with the increased complex formation between Bax and Bcl-2 (Figure 5C). We therefore investigated if Bcl-2 overexpression confers protection against fucoidan-induced apoptosis in U937 cells. The results of our study indicate that ectopic Bcl-2 overexpression can block fucoidan-induced caspases activation, cleavage of Bid, as well as PARP and DNA fragmentation to inhibit fucoidan-mediated apoptosis (Figure 7A–C). Because strategies to overcome Bcl-2-mediated resistance to apoptosis have the potential to greatly increase treatment efficacy, we next examined the ability of a small molecule Bcl-2 inhibitor, HA14-1 [36], which prevents Bcl-2 interaction with Bax, leading to apoptotic cell death [37], to determine if it could reverse the anti-apoptotic effect of Bcl-2 and enhance fucoidan-mediated apoptosis. As Figure 7D shows, Bcl-2 overexpression U937 cells synergistically incubated with HA14-1 and fucoidan had significantly greater levels of apoptotic cells than those treated with fucoidan alone, indicating a close relationship of Bcl-2 to the resistance of fucoidan-mediated apoptosis.

Taken together, these results suggest that activation of p38 MAPK signaling pathway participate in fucoidan-induced apoptosis and that reduction in MMP through modulation of Bcl-2 family proteins is important in fucoidan-induced apoptosis in U937 cells.

## 3. Experimental Section

### 3.1. Reagents

Fucoidan (Product F5631, isolated from *F. vesiculosus*) [38], 3-(4,5-dimetylthiazol-2-yl)-2,5-diphenyl-tetrazolium (MTT), propidium iodide (PI), JC-1 and DAPI were purchased from Sigma-Aldrich (St. Louis, MO, USA). Fetal bovine serum (FBS) and caspase activity assay kits were obtained from GIBCO-BRL (Gaithersburg, MD, USA) and R & D Systems (Minneapolis, MN, USA), respectively. ERK-specific inhibitor, PD98059, JNK-specific inhibitor, SP600125, and p38 MAPK-specific inhibitor, SB203580, were purchased from Calbiochem (San Diego, CA, USA). DNA staining kit (CycleTEST™ PLUS Kit, San Jose, CA, USA) and ECL kit were purchased from Becton Dickinson (San Jose, CA, USA) and Amersham (Arlington Heights, IL, USA), respectively. All antibodies were purchased from Santa Cruz Biotechnology (Santa Cruz, CA, USA). 

### 3.2. Cell Lines and Cell Culture

The human leukemic U937 HL60, K562, and THP1 cells were purchased from the American Type Culture Collection (Rockville, MD, USA), and maintained at 37 °C in a humidified 95% air and 5% CO_2_ in RPMI1640 supplemented with 10% heat-inactivated FBS, 2 mM glutamine, 100 U/mL penicillin, and 100 μg/mL streptomycin. Bcl-2 overexpressing U937 cells, a generous gift from T. K. Kwon (Department of Immunology, Keimyung University, School of Medicine, Taegu, Korea), were maintained in a medium containing 0.7 μg/mL geneticin (G418 sulfate, Calbiochem). Fucoidan was dissolved in phosphate buffered saline (PBS) as a stock solution at a 200 mg/mL concentration, and the stock solution was then diluted with the medium to the desired concentration prior to use.

### 3.3. MTT Assay

To investigate the cell viability, the cells were seeded in 6-well plates at a density of 2.5 × 10^5^ cells per well and stabilized for 24 h. The cells were then treated with various concentrations of fucoidan for the desired times. MTT working solution (0.5 mg/mL) was then added to the culture plates and incubated continuously at 37 °C for 48 h. The culture supernatant was completely removed from the wells, and DMSO was added to completely dissolve the formazan crystals. The absorbance of each well was measured at a wavelength of 540 nm with a microplate reader (Molecular Devices, Palo Alto, CA, USA). The effect of fucoidan on the inhibition of cell growth was assessed as the percentage of cell viability, where the vehicle-treated cells were considered 100% viable.

### 3.4. Measurement of Cell Cycle and MMP by a Flow Cytometer

For analysis of the cell cycle, cells were collected, washed with cold PBS, and fixed in 75% ethanol at 4 °C for 30 min. The DNA content of the cells was measured using a DNA staining kit according to the manufacturer’s instructions. Then flow cytometric analyses were carried out using a flow cytometer and the relative DNA content determined using CellQuest software based on the presence of red fluorescence. The MMP (ΔΨm) was determined using the dual-emission potential-sensitive probe, JC-1. The cells were collected and incubated with 10 μM JC-1 for 20 min at 37 °C, in the dark. The cells were then washed once with PBS and analyzed by a flow cytometer [39]. 

### 3.5. DNA Fragmentation Assay

After the fucoidan treatment, the cells were lysed in a buffer containing 10 mM Tris-HCl, pH 7.4, 150 mM NaCl, 5 mM EDTA, and 0.5% Triton X-100 for 1 h at room temperature. The lysates were vortexed and cleared by centrifugation at 19,000× *g* for 30 min at 4 °C. The DNA in the supernatant was extracted using a 25:24:1 (v/v/v) equal volume of neutral phenol:chloroform:isoamyl alcohol (Sigma-Aldrich, St. Louis, MO, USA) and analyzed electrophoretically on 2.0% agarose gels containing 0.1 μg/mL EtBr (Sigma-Aldrich).

### 3.6. DAPI Staining

After a treatment of fucoidan, the cells were harvested, washed in ice-cold PBS, and fixed with 3.7% paraformaldehyde (Sigma-Aldrich) in PBS for 10 min at room temperature. The fixed cells were washed with PBS and stained with a DAPI solution for 10 min at room temperature. The cells were washed two more times with PBS and analyzed via a fluorescence microscope (Carl Zeiss, Oberkochen, Germany).

### 3.7. Protein Extraction and Western Blotting

Cells were harvested and washed twice in PBS at 4 °C. Total cells lysates were lysed in lysis buffer (40 mM Tris (pH 8.0), 120 mM, NaCl, 0.5% NP-40, 0.1 mM sodium orthovanadate, 2 μg/mL aprotinin, 2 μg/mL leupeptin, and 100 μg/mL phenymethylsulfonyl fluoride). The supernatants were collected and protein concentrations were then measured with protein assay reagents (Pierce, Rockford, IL, USA). Equal amounts of protein extracts were denatured by boiling at 95 °C for 5 min in sample buffer (0.5 M Tris-HCl, pH 6.8, 4% SDS, 20% glycerol, 0.1% bromophenol blue, 10% β-mercaptoethanol) in a ratio of 1:1, subjected to 6%–15% SDS-polyacrylamide gels, and transferred to polyvinylidene difluoride membranes (Schleicher & Schuell, Keene, NH, USA) by electroblotting. The membranes were blocked with 5% non-fat dry milk in PBS with Tween 20 buffer (PBS-T) (20 mM Tris, 100 mM NaCl, pH 7.5, and 0.1% Tween 20) for 1 h at room temperature. Membranes were then incubated overnight at 4 °C with the primary antibodies, probed with enzyme-linked secondary antibodies, and visualized using an ECL kit according to the manufacturer’s instructions.

### 3.8. Caspase Activity Assay

The activities of caspases were determined by colorimetric assay kits, which utilize synthetic tetrapeptides (Asp-Glu-Val-Asp (DEAD) for caspase-3; Ile-Glu-Thr-Asp (IETD) for caspase-8; Leu-Glu-His-Asp (LEHD) for caspase-9, respectively) labeled with p-nitroaniline (pNA). Briefly, fucoidan-treated and untreated cells were lysed in the supplied lysis buffer. The supernatants were collected and incubated with the supplied reaction buffer containing DTT and DEAD-pNA, IETD-pNA, or LEHD-pNA as substrates at 37 °C. The reactions were measured by changes in absorbance at 405 nm using the VERSAmax tunable microplate reader. 

### 3.9. Co-Immunoprecipitation Assay

The binding activity of proteins was determined by co-immunoprecipitation (Co-IP) assay. For this study, total cell lysates were incubated with the desired antibodies for 1 h at 4 °C and the immuno-complex was collected on protein A-Sepharose beads (Sigma-Aldrich) for 1 h and washed 5 times with lysis buffer prior to boiling in SDS sample buffer. Immunoprecipitated proteins were separated on SDS-polyacrylamide gels and transferred to nitrocellulose membranes for Western blot analysis [40].

### 3.10. Statistical Analyses

All data are presented as mean ± SD. Significant differences among the groups were determined using the unpaired Student’s *t*-test. A value of *p < 0.05 was accepted as an indication of statistical significance. All the figures shown in this article were obtained from at least three independent experiments.

## 4. Conclusions

In conclusion, this study demonstrates that fucoidan significantly induces apoptosis by activation of caspases, loss of MMP, Bax translocation to mitochondria from cytosol, and release of cytochrome *c* from mitochondria to cytosol via activation of p38 MAPK in human leukemia U937 cells, providing that important mechanistic insights related to p38 MAPK-mediated apoptotic cell death by fucoidan in U937 cells. In addition, Bcl-2 overexpression inhibits fucoidan-induced apoptosis and the small molecule inhibitor of Bcl-2 restores the blockage of apoptosis by Bcl-2 overexpression, indicating the need for future studies to better define the optimal combinations of fucoidan and Bcl-2 inhibitors to overcome Bcl-2-mediated resistance, thereby increasing therapeutic efficacy, in additional models of leukemia and other cancers.
